# A Novel Kinetic Method to Measure Apparent Solubility Product of Bulk Human Enamel

**DOI:** 10.3389/fphys.2017.00714

**Published:** 2017-09-21

**Authors:** Linda Hassanali, Ferranti S. Wong, Richard J. M. Lynch, Paul Anderson

**Affiliations:** ^1^Dental Physical Sciences Unit, Institute of Dentistry, Queen Mary University of London London, United Kingdom; ^2^Innovation Research and Development, Oral Healthcare, GlaxoSmithKline Weybridge, United Kingdom

**Keywords:** enamel, demineralization, solubility, calcium, phosphate, scanning microradiography, solubility product

## Abstract

**Introduction:** Tooth enamel mineral loss is influenced by its solubility product value, which is fundamental to the understanding of de- and remineralization resulting from a carious or erosive challenge. Published pKsp values for human enamel and hydroxyapatite range from 110 to 126 suggesting a heterogeneous nature of enamel solubility. However, this range of values may also result from the variety of methods used, e.g., some authors reporting values for suspensions of enamel powder and others for bulk enamel. The aim of this study was to develop a method to measure the solubility of bulk human enamel under controlled *in vitro* conditions simulating demineralization behavior of enamel within the oral environment using scanning microradiography (SMR). SMR was used to monitor real-time changes in enamel demineralization rates at increasing calcium concentrations in a caries simulating demineralization solution until the concentration at which thermodynamic equilibrium between enamel and solution was achieved.

**Method:** 2 mm thick caries free erupted human enamel slabs with the natural buccal surfaces exposed were placed in SMR cells exposed to circulating caries-simulating 2.0 L 0.1 M pH = 4.0 acetic acid, at 25°C. SMR was used to continuously measure in real-time the decrease in mineral mass during the demineralization at 5 different points from on each slab. Demineralization rates were calculated from a linear regression curve of projected mineral mass against demineralization time. Changes in the demineralization rates were monitored following a series of successive increases in calcium (and phosphate at hydroxyapatite stoichiometric ratios of Ca:P 1.67) were added to the demineralizing solution, until demineralization ceased. The pH was maintained constant throughout.

**Results:** Demineralization halted when the calcium concentration was ~30 mM. At higher calcium concentrations, mineral deposition (remineralization) occurred. By comparison with results from speciation software calculations for the calcium phosphate ternary system, this result suggests that the bulk solubility product of enamel (pKsp_BEnamel_) under the conditions used is 121.

**Discussion:** The apparent pKsp_BEnamel_ under these conditions was higher than many previous reported values, and much closer to those previously reported for HAp. However, this is a bulk value, and does not reflect that enamel is a heterogeneous material, nor the influence of ionic inclusions.

## Introduction

Tooth decay is a multifactorial process which is influenced by genetic and environmental factors (Keyes, 1962; Borutta et al., 2010). For example, caries related illnesses have become a cause for concern within the UK according to a recent LGA 2013 report (Brunton, 2014; Local Government Authority Report, 2016). Such factors include those associated with the host tissue such as the structure and composition of enamel (Robinson et al., 1995), the pellicle and saliva (Leung and Darvell, 1991) as well as environmental factors such as diet and socioeconomic status (Lalloo and Myburgh, 1999; Hobdell et al., 2003). The kinetics of mineral loss and precipitation is influenced by many factors (Gibbs, 1873; Arends, 1981; DeVoe, 2016), including its physical (Nancollas, 2005) and chemical structure, and the composition and pH, and, also the chemical equilibria between enamel and solution (Dorozhkin, 2012), i.e., the solubility product constant (Ksp). Thus, the dissolution of bulk enamel is significantly influenced by its solubility product (which is defined as the mathematical product of its ion activities raised to the power of their stoichiometric coefficients). Enamel is a calcium deficient form of HAp (Elliott et al., 1994). The Ksp of HAp (Ksp_HAp_) is defined, from the stoichiometry:

(1) $${\text{Ca}}_{10}{\left({\text{PO}}_{4}\right)}_{6}{\text{OH}}_{2}⇆10{\text{Ca}}^{2+}+6{\text{PO}}_{4}^{3-}+2{\text{OH}}^{-}$$

and therefore:

(2) $${\text{Ksp}}_{\text{HAP}}={\left\{{\text{Ca}}^{2+}\right\}}^{10}{\left\{{\text{PO}}_{4}^{3-}\right\}}^{6}{\left\{{\text{OH}}^{-}\right\}}^{2}$$

where {} denotes ionic activities at equilibrium raised by the power according to the stoichiometry. In this study the negative logarithm to the base 10 of the Ksp is used (pKsp).

Solubility is the propensity for a solute to dissolve in a solvent and arrive at an end point where the potential energy of the system is at its lowest (Smith, 2015). pKsp is a function of the amount of dissolved solid in solution at equilibrium. Table 1 shows published pKsp values for bulk enamel, and for HAp, which are within the range 110–126. This range suggests uncertainties in the precise value, and as to whether the solubility of bulk enamel is similar to that of HAp, and, if it is influenced by chemical inclusions. The range also suggests incongruent dissolution behavior of both enamel and hydroxyapatite. However, the range in the measured pKsp values of both enamel and HAp also suggests that the values may also be dependent on the choice of experimental protocol, such as the types of materials used (e.g., whether powdered or bulk samples); the methodology; and the analysis/calculations used to calculate the pKsp value.

**Table 1:** Published pKsp_enamel/HAp_ values with references and summary of methodologies.

**pKsp**	**References**	**Substrate and method**
106–116	Patel and Brown, 1975	Human powdered enamel. pH range 4.5–7.6. Measured the amounts dissolved to calculate Ksp.
110 *(Apparent* solubility product 116)	Zhang et al., 2000	Contact Microradiography assessed the demineralization of sections of enamel in lactic acid solutions ranging pH 5–5.07 over a range of DS_en_ values (0.28–0.79) that were based on a pKsp_enamel_ of 110. Suggest that enamel may have an *apparent* solubility product of 116 based on experimental findings.
116	Shellis et al., 1993	Enamel powder equilibrated with 4 or 17 mM of H_3_PO_4_ at 37°C. Amounts dissolved were measured and used to calculate the ion activity product at equilibrium = Ksp.
117	Dawes, 2003	HAp Ksp cited in special feature article
118	McDowel et al., 1977	4 g synthetic hydroxyapatite. Ion concentrations at equilibrium then measured. Ksp determined as a function of temperature.
Enamel: 117.6 HAp: 121.82 (Pooled from pH 4.5–5.5)	Shellis and Wilson, 2004	Powdered synthetic HAp. Premolar enamel powder. 5 mg equilibrated with acetic buffers at pH 4.5, 5.0 (0.15 M) 5.5 (0.2 M) in a range of degrees of saturation (range of pI_HA_) at 37°C. Mass fraction dissolved plotted against pI_HA_ to give distribution curves at each pH. No statistical difference in pH differences.
126	Pan and Darvell, 2007	Solid HAP titrated with KCl solutions at pH 3.2, 3.6, and 4.1 at 37°C. Dissolution monitored using a semiconductor-diode laser scattering system that peaked each time solid was added and disappeared when all solid dissolved. Same solution was adjusted to a decreasing pH using 1M HCl.

Conventional methods of the measurement of solubility product of enamel use chemical equilibrium conditions, with the concentration of solute in the solution at saturation determined by an analytical procedure (Chen et al., 2004; Brittain, 2014). In this study, the aim was to develop a method to measure the rate of bulk enamel demineralization at increasing calcium concentrations in the demineralizing solution (and therefore increasing degrees of saturation) until dissolution stopped and equilibrium achieved. No chemical analyses of solutions was required.

The dissolution rate of enamel can be expressed as a function of the degree of saturation with respect to enamel (Margolis and Moreno, 1985, 1992):

(3) $$R=k\bar{\text{A}}{\left(C_{\text{eq}}-C_{\text{b}}\right)}^{\text{n}}$$

where;

R = rate;

k = rate constant;

$\overset{-}{\text{A}}$ = specific surface area of dissolving surface;

C_eq_ = equilibrium concentration of solvent;

C_b_ = undersaturated concentration of solvent;

n = integer.

Equation (3) indicates that the addition and/or presence of ionizable solutes will influence the rate (Berner, 1978). It is therefore important to ensure that all ionic concentrations are accounted for and controlled. Previous studies have highlighted that caution is required to ensure that the monitoring device used to measure dissolution rates is sensitive enough to identify the point at which equilibrium is reached, and must not mistake extremely low rates that are undetectable for the condition of equilibrium (Zhang et al., 2000).

In this study, a real-time methodology scanning microradiography (SMR) was used to accurately measure the rate of bulk enamel mineral loss in demineralizing solutions that contained increasing concentration of calcium. The calcium concentration at which the rate ceased (i.e., equilibrium) was then compared with calculated results of degree of saturation at a range solubility products of hydroxyapatite. These values were calculated using a ionic speciation program (Chemist) incorporating the chemical conditions used in the SMR experiment, to calculate the different species that would be present in the dissolution media. The SMR methodology provides a precise means of measuring real-time dissolution of enamel blocks under controlled conditions that simulate caries. Further, the methodology is directly quantitative achieved using an X-ray photon counting system with minimum error (for a full description of SMR and calculation of statistical errors of the photon counting system see Anderson et al., 1998).

For the solubility product to be accurately calculated, all phases and ion-pairs present during dissolution need to be accounted for. Chemical speciation of a solution describes the chemical form and concentrations of each species present, and can be derived using the thermodynamic principals of mass balance (Quinn and Taylor, 1992). Speciation software can be used to calculate the chemical speciation of complex systems. In this study, we describe a novel kinetic method to measure the *apparent*-pKsp_BEnamel_ under conditions relevant to caries, in conjunction with a speciation program so that the *apparent*-pKsp_BEnamel_ value could be calculated at conditions of pH = 4.0 and 25°C. The rate of demineralization of natural unaltered surfaces of human permanent enamel blocks was measured using SMR to determine the effective solubility in an inorganic caries-like demineralizing solution with a decreasing degree of undersaturation with respect to HAp.

## Materials and methods

### Scanning microradiography

SMR is an X-ray absorption technique that enables the monitoring of mineral loss (and eventually gain once equilibrium is surpassed) in thin sections of enamel samples during demin/remineralization studies (Anderson et al., 1998). Developed to overcome some of the limitations of conventional (contact) microradiography which required samples to be in contact with a photographic film, SMR can measure mineral changes in real time as solutions can be circulated through SMR cells, within which samples are mounted, at a controlled rate simulating salivary flow.

**Figure 1:**
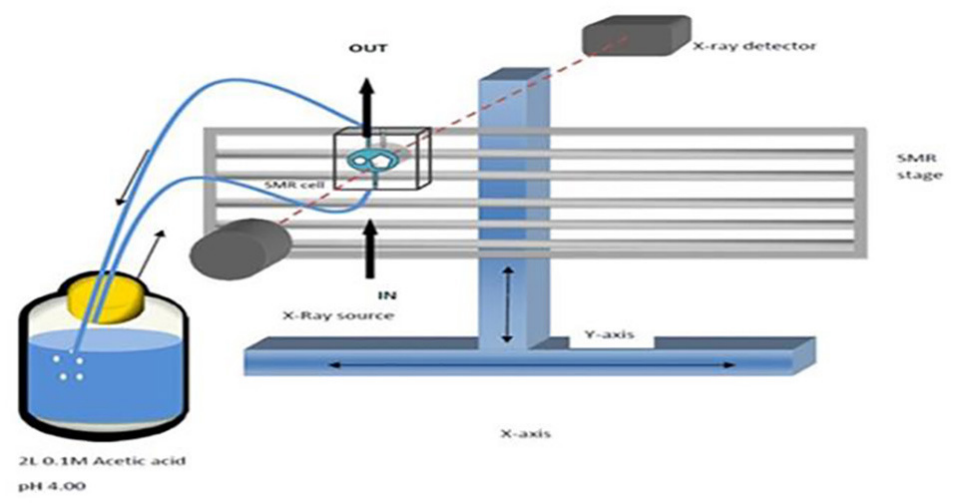
Schematic diagram of SMR experimental set up showing X-rays parallel to the direction of acid attack and perpendicular to the enamel surface.

In this study, the integrated mode of SMR (Figure 1) was used whereby the direction of acid attack was parallel to a 15 μm diameter X-ray beam so that changes in the projected mineral mass from the surface could be measured as mineral was lost from the surface and receded to the enamel dentine junction (Anderson and Elliott, 2000). The X-ray generator was operated at a maximum voltage of 41 kV and a current of 0.7 mA. The transmitted X-ray intensity for energies selected at 22.1 keV at each point was measured and the mass of absorbing mineral determined. Since demineralization is nearly linear with time under constant chemical conditions (Elliott et al., 1994; Wang et al., 2006), the rate of projected mineral mass loss of the same enamel sample at decreasing degrees of undersaturation with respect to HAp could be measured (Margolis et al., 1999).

### Enamel sample preparation for SMR

Eleven different permanent enamel blocks were prepared from teeth extracted for orthodontic purposes, with the roots removed and discarded and stored in methylated ethanol solution at room temperature were analyzed. Teeth were cut parallel to the tooth face into 2 mm enamel blocks with the natural buccal surfaces intact using a diamond cutting saw (Microslice 2, Malvern Instruments, UK). Ethical approval was granted by Queen Mary Research Ethics Committee (QMREC 2011/99).

**Figure 2:**
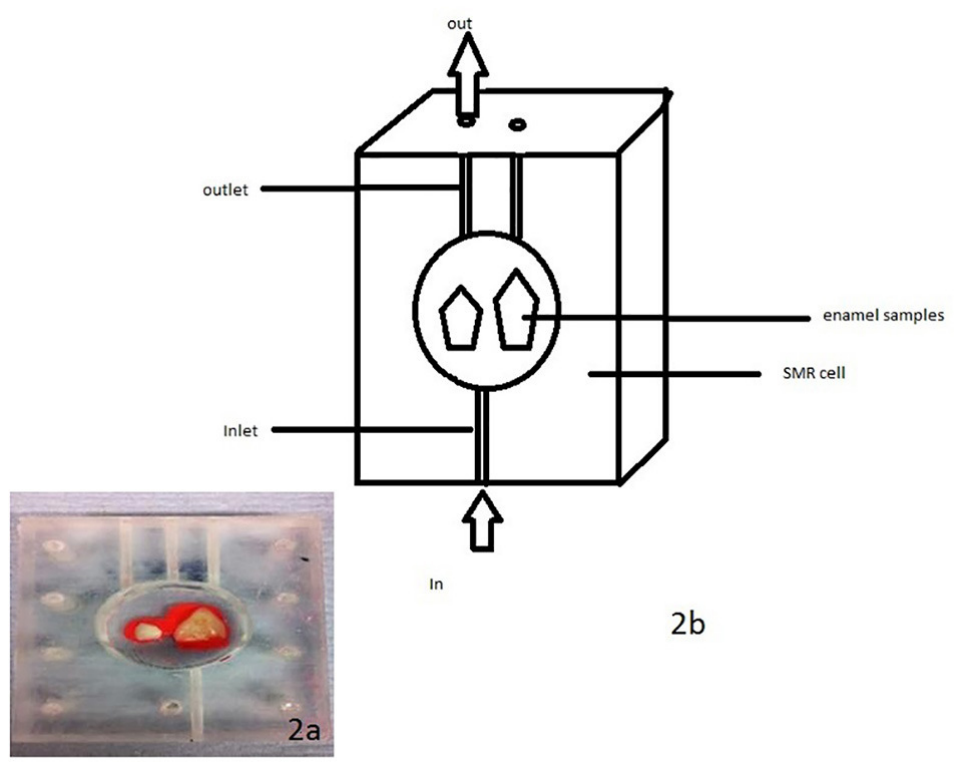
**(a,b)** A photo and schematic diagram of human enamel blocks in an SMR cell with figure **(b)** showing the flow of acid into and out of the cell.

The enamel blocks were mounted in SMR cells with the natural surfaces exposed (Figure 2). Each was scanned with X-ray Microtomography (XMT) (Elliott et al., 1994) to identify caries free (unaffected) areas suitable for analysis with SMR. The SMR cells were then mounted on the XY stage of the SMR apparatus. The samples were initially immersed in deionized water circulated using a peristaltic pump as previously described in Mishra et al. (2009) for 48 h in order to ensure the samples were fully hydrated prior to the commencement of the dissolution experiment. The temperature was maintained at 25.0 ± 1.0°C throughout the experiment, as solubility is highly dependent on temperature.

### Calculation of integrated mineral mass using SMR

The mineral mass of enamel was calculated using the mass absorption coefficient (μ_m_) of pure hydroxyapatite as previously described in Anderson et al. (1998), for AgKα radiation at 22.1 keV (4.69 cm^2^ g^−1^). The integrated mineral mass per unit area (m) at each data point is:

(4) $$m=\frac{1}{\begin{array}{c} \mu_{m}\\ \; \end{array}}\ln\frac{No}{N}$$

where;

μ_m_ is mass attenuation coefficient of HAp for AgKα radiation at 22.1 keV.

N_0_ is number of incident photons.

N is number of transmitted photons.

### Identification of SMR scanning points

Before the dissolution experiment, SMR area scans were carried out in order to locate the samples on the XY stage, and to select the coordinates of ~5 points suitable for measuring mineral mass changes as identified using XMT (see section Enamel Sample Preparation for SMR). The selected points were located horizontally across the buccal enamel surface from distal to mesial.

### Preparation of demineralization solution

2.0 L of pH = 4.0 acetic acid 0.1 M (Anderson et al., 2004) was prepared using 12 g of pure acetic acid, diluted with deionized water and adjusted to a pH of 4.0 with a 1.0 M stock solution of KOH using a pH meter (Orion-pH/ISE meter Model 710).

### Calculation of demineralization rates

**Figure 3:**
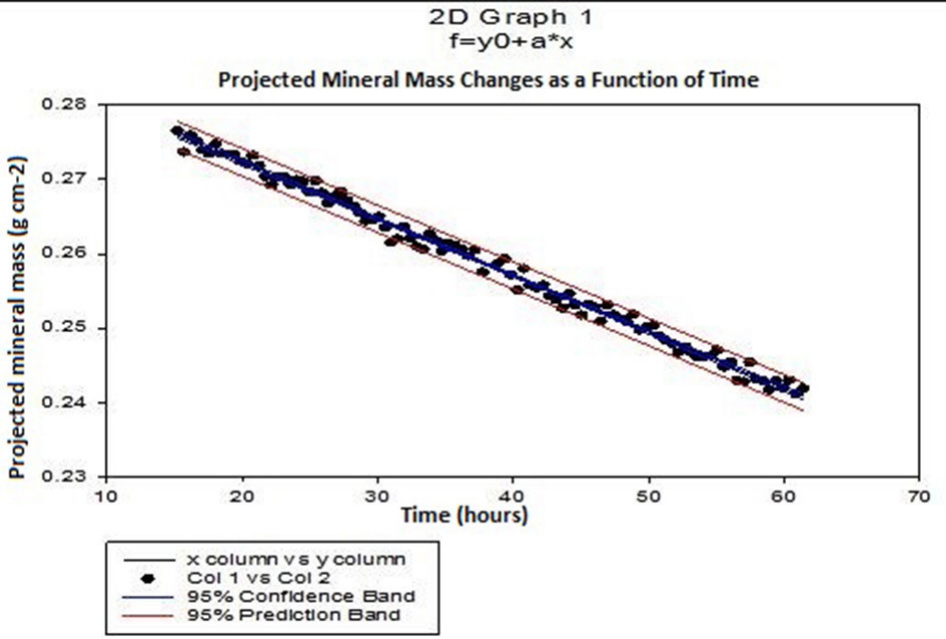
Typical SMR showing the linear changes in projected mineral mass of enamel with time and the 95% confidence intervals (*p* ≤ 0.0001) in 0.1 M acetic acid solution at pH = 4.0.

Mean demineralization rates were calculated using linear regression from the projected mineral mass curves obtained from SMR analysis of selected points on the enamel surface (Figure 3). The rate of mineral loss was determined from the gradient in units of grams per unit exposed area per hour. Typical times between successive data points on the same sample was about 10 min.

### Calculation of equilibrium conditions

Thermodynamic equilibrium was assumed to be when demineralization rate was zero. The calcium concentration at thermodynamic equilibrium was determined by plotting demineralization rates against calcium concentration (**Figure 6**) and determining the x-intercept (calcium concentration) from the line equation of the polynomial regression curve (of order 2) using MATLAB (MathWorks).

### Preparation of calcium and phosphate increments

0.66 g increments of CaCl_2_ and 0.822 g increments of K_2_HPO_4_ were weighed so that any additions of each increment into the 2.0 L of acetic acid solution would give a concentration of 3.0 mM calcium and 1.8 mM phosphate (Ca/P 1.67).

The concentration of calcium and phosphate in the demineralizing solution was increased incrementally by 3.0 mM calcium and 1.8 mM phosphate, respectively (Ca/P 1.67) every 48 h. Steps were taken to ensure pH was maintained constant throughout. The rate of demineralization of the enamel sample was measured at each increase in calcium/phosphate concentration increment using SMR.

## Calculation of degree of saturation of demineralizing solutions used

**Figure 4:**
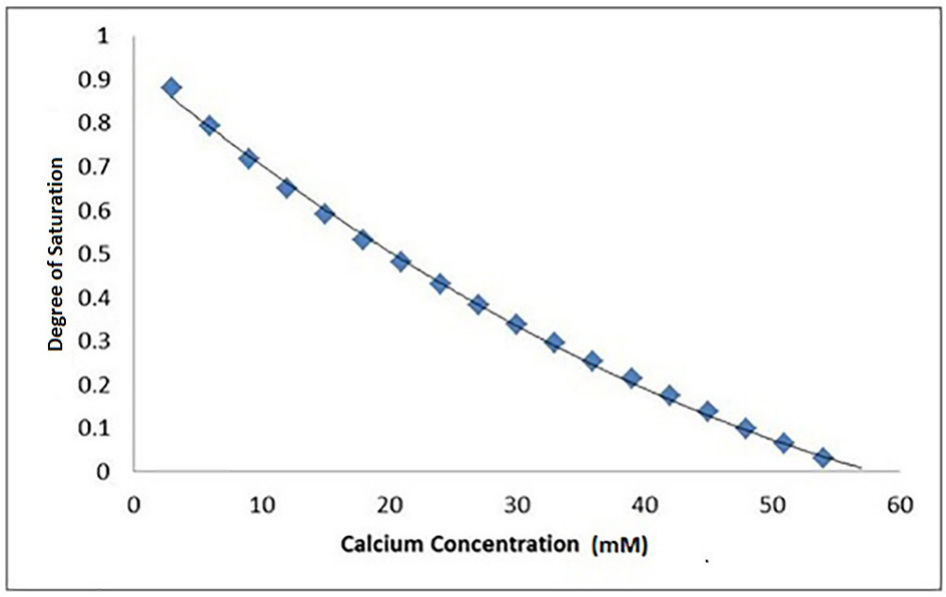
A typical Chemist plot for a pKsp of 118 showing thermodynamic equilibrium is reached at 57 mM of calcium at the x-intercept, under pH = 4.0 at 25°C conditions. The degree of saturation with respect to HAp was calculated based on the conditions and calcium and phosphate concentrations used in the SMR experiment.

**Figure 5:**
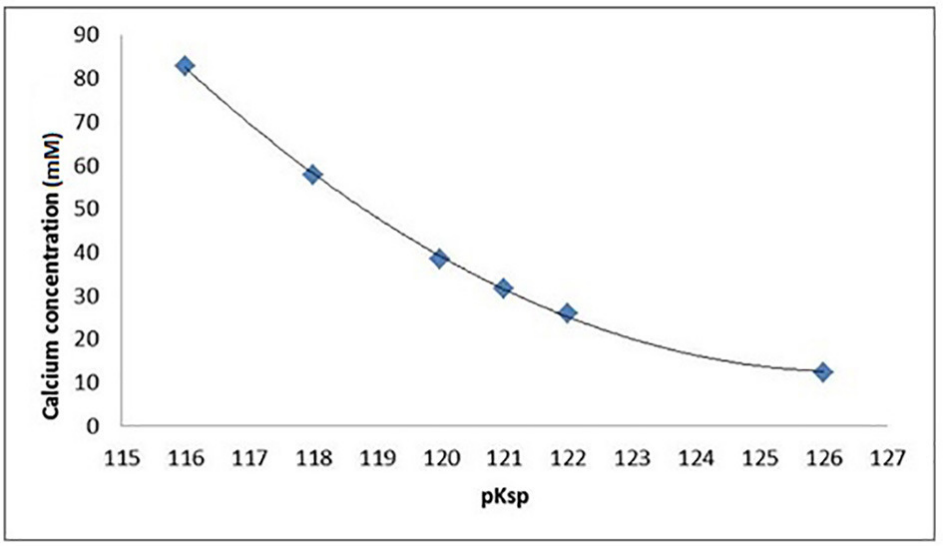
Calcium and phosphate concentration (mM) in equilibrium with HAp as a function of the pKsp assumed for HAp, assuming pH = 4.0, 0.1 M acetic acid, and 25°C as calculated by Chemist.

An ionic speciation program, Chemist (MicroMath, Missouri, USA) was used to calculate the degree of saturation with respect to hydroxyapatite (DS_HAp_) for a solution at pH = 4.0 in a range of solutions with increasing calcium concentrations identical to those used in the SMR measurements. This calculation was repeated for a range of pKsp values from 116 to 126, and the degree of saturation as a function of calcium concentration was then plotted for each pKsp value (Figure 4). The calcium concentration at equilibrium (i.e., when the saturation was 1) was determined from each plot, and then these were then plotted for each pKsp value (Figure 5). The corresponding calcium concentration required to halt demineralization from the SMR data (Figure 3) was then used to determine the pKsp for the calcium concentration required to reach equilibrium (**Figure 7**).

## Results

### Enamel demineralization rate as a function of increasing calcium concentration

Figure 3 shows a typical plot of mineral mass changes of bulk enamel with time during demineralization with 0.1 M acetic acid at pH = 4.0 at 25°C (confidence intervals of 95%, *p* ≤ 0.0001). Similar results showing a linear rate of mineral loss with time were obtained at each incremental additional calcium concentration. Mean enamel demineralization rates were calculated from a total of 33 points on 11 different permanent enamel blocks (see section Calculation of Demineralization Rates for calculation of demineralization rates procedure).

**Figure 6:**
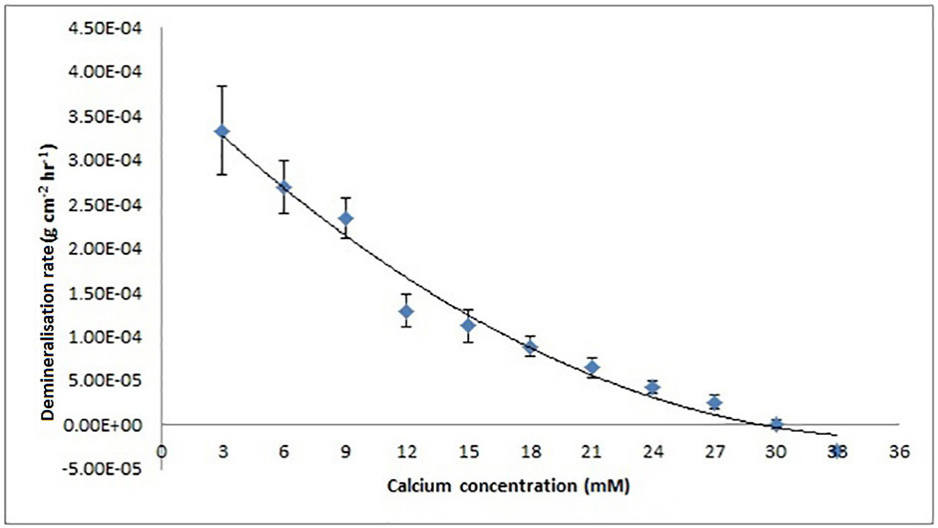
Enamel demineralization rate as a function of increasing calcium concentration at pH = 4.0 and 25°C based on results from the SMR experiment. Rate is zero at a calcium concentration of 30 mM calculated using MATLAB from the x-intercept of the regression curve (*R*^2^ = 9.8).

The mean demineralization rates were then plotted as a function of calcium concentration (Figure 6), which showed a decreasing, but non-linear, trend (SE ranged from ± 2.90 × 10^−06^ to ± 1.09 × 10^−05^). This result shows that under the conditions used, the calcium concentration required to achieve thermodynamic equilibrium was 30 mM as determined from the horizontal intercept (regression curve *R*^2^ = 0.98), calculated using MATLAB.

### Identifying the pKsp of bulk enamel using ion speciation software

**Figure 7:**
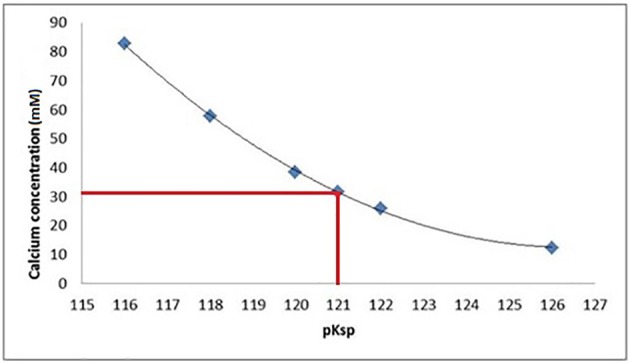
At a pKsp of 121, a calcium concentration of 30 mM is required for saturation of a solution with respect to HAp at pH = 4.0.

Data fitting of the SMR data shows that calcium concentration required for demineralization of bulk enamel to cease (at conditions of pH = 4.0 and 25°C), i.e. that equilibrium is achieved is 30 mM. under the same conditions. Fitting to the speciation software data suggests a pKsp of 121 corresponds to a calcium concentration of 30 mM to reach thermodynamic equilibrium for pH = 4.0 and 25°C conditions. This result suggests that the pKsp_BEnamel_ is 121 (Figure 7).

## Discussion

It is important to measure the solubility of enamel under caries like conditions using a precise measuring system in order to accurately derive the solubility product. This information is relevant to the development of our understanding of caries and erosion and to develop preventative measures such as to screen anticaries agents.

The SMR data showed that the bulk human enamel demineralization at pH = 4.0 was reduced to zero at a calcium concentration of 30 mM. Correlation with the speciation software calculations shows this corresponds to a pKsp value of 121. Figures 4, 6 show a consistency between the speciation software of degree of saturation (proportional to chemical driving force) and the SMR kinetic data, indicating that the method was appropriate.

Figure 6 shows that the decrease in demineralization rate was non-linear (rather than linear as would be expected from simple first-order dissolution kinetics) similar to that obtained from speciation calculations for all pKsp values (Figure 4). This is consistent with what would be predicted for the calcium phosphate ternary system (Leaist et al., 1990).

As discussed earlier it is important to account for all possible phase transformations, and equilibria, as a lack of information on the resulting equilibria results in imperfect calculations (Pan and Darvell, 2007). Ion speciation programs rely on databases that report experimental results for speciation constants as well as the methods and conditions of the experiments reported (VanBriesen et al., 2010). The similarities observed in the data from the SMR method and the speciation software (Figures 4, 6 respectively) confirm the methodology. The small standard errors (Figure 6) also indicate that there was little variation in the demineralization rates between samples.

The *apparent*-pKsp_BEnamel_ value of 121 measured in this study is higher than many previously reported values (see Table 1), and higher than many values reported for pure HAp. As mentioned above, the methodology may influence the values reported for enamel and hydroxyapatite (Dorozhkin, 2012, Liu et al., 2013).

**Figure 8:**
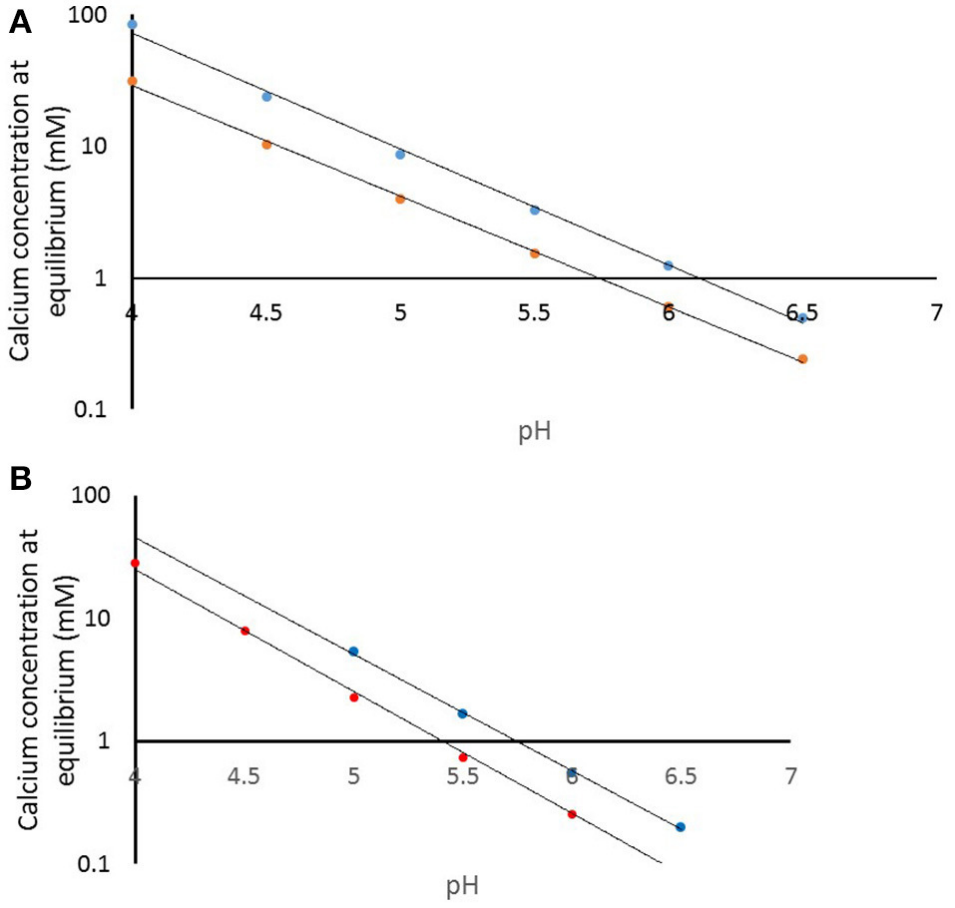
**(A)** Calculated calcium concentration required for equilibrium at pKsp values of 116 (blue) and 121 (red) for a demineralization solution of 100 mmol/L acetic acid. **(B)** Calculated calcium concentration required for equilibrium at pKsp values of 116 (blue) and 121 (red) with only that acid or base required for pH condition.

How does this impact on the clinical situation? We have used the speciation software to model the equilibrium conditions as a function of pH for the pKSp values under the conditions used in the experiment. Figure 8A shows the calcium concentration required for equilibrium under the conditions used, including acid concentration and temperature (plotted on a log scale) for a pKSp value of 116 (blue), and for pKsp of 121 (red). This shows that at pH 4.0, the calcium concentration required for equilibrium for a pKSp = 116 would be 85 mM, whereas for pKSp = 121 this would only be 31 mmol/L. However, let us consider a calcium concentration of 1 mmol/L, cited as the value of free calcium concentration in saliva (Lagerlof, 1983). Then, from Figure 8A, this would suggest that the saturation would occur only at pH above 6.0. This is often called the “critical pH” (Dawes, 2003). However, using a value of pKSp of 121, then this would suggest a much lower “critical pH” (at a calcium concentration of 1 mM). Of course, these calculations were performed with an acid concentration of 0.1 M, which is high for oral conditions. Figure 8B is a repeat of this calculation, but with no fixed acetic concentration (and is therefore not necessarily repeatable in a laboratory), but represents the opposite extreme with a zero acetic concentration. This reduces the critical pH value, assuming a free calcium ion concentration of 1.0 mM (Lagerlof, 1983). The oral environment acid concentration is likely to be somewhere between these extremes, but this calculation confirms that a pKSp value of 116 is too low, and that is likely to be nearer to 121, otherwise there would be insufficient calcium in the oral environment to prevent enamel being undersaturated. It is also likely that other factors including salivary proteins (Kosoric et al., 2010) also play in role in the protective function of enamel, and will modify the apparent solubility product.

Whilst these results provide an insight into the dynamics of enamel dissolution under pH = 4.0 conditions at 25°C, the result is for bulk enamel only, and based on an *in vitro* design, and so it is important to acknowledge that any conclusions made are limited to these conditions only. For instance, further similar SMR studies are required to determine the effect pH (Ito et al., 1996). An increase in dissolution rate is observed when pH is reduced (Gao et al., 2001). Furthermore, published data on the effect of pH on the solubility product of enamel is contradictory, e.g., Shellis and Wilson (2004) found no statistical difference in the solubility product of powdered enamel at different pH values between 4.5 and 5.5 whereas the earlier studies of Patel and Brown (1975) reported lower solubility product values of 106–116 over a pH range of 4.5–7.6. The inconsistency in results is in spite of both experiments using powdered enamel as the substrate which again highlights the advantage of using bulk enamel as the substrate rather than powdered. Such measurements will also confirm or otherwise the marked change in slope of the solubility isotherm for HAp at around pH 3.9 as reported by Pan and Darvell (2009a,b).

Also, further similar SMR studies are required to study the influence of different ionic substitutions on both enamel and HAp powder, including carbonate, Mg^2+^, F^−^, etc. would provide significant further information on the chemistry of demineralization process. Also, similar studies are required too for a comparison of bulk enamel values with values obtained for powdered hydroxyapatite, similar to the studies reported in Table 1.

Further, the physical and chemical heterogeneities within enamel should not be ignored (Arends and Jongebloed, 1977; Zhang et al., 2000; Bechtle et al., 2012). For example, demineralization rates of prismatic, interprismatic and aprismatic enamel are not the same due to differences between the organization of crystals, the presence of more soluble material, and the porosity (Avery et al., 1961; Boyde, 1967; Shellis, 1996; Shellis and Dibdin, 2000). On a chemical compositional level, enamel is a substituted calcium hydroxyapatite (Neel et al., 2016). Its composition varies with ions such as F^−^, ${\text{CO}}_{3}^{2-}$ and Mg^2+^ replacing OH^−^, ${\text{PO}}_{4}^{3-}$ and Ca^2+^ within the stoichiometry allegedly altering the solubility of enamel (Aoba, 1997; Elliott, 2003; West and Joiner, 2014; Liu et al., 2016). Thus, the site of source material may be a critical factor. Further solubility measurements are also needed to investigate the influence of the structural or chemical heterogeneities of enamel on the demineralization rate. As the enamel is etched away and moves toward the enamel dentine junction there are changes in demineralization rate resulting from gradients in ionic substitutions within enamel structure (Anderson and Elliott, 2000). In addition, within enamel, the processes of demineralization and remineralization may not be co-localized, with ions diffusing in different directions (Anderson and Elliott, 1992).

In conclusion, the SMR method described here provides greater insight into bulk enamel dissolution by measuring the *effect* of calcium concentration on the dissolution kinetics of bulk enamel demineralization under standardized caries-like conditions. The measured pKsp_BEnamel_ value of ~121 is similar to that reported by Shellis and Wilson (2004) for pure HAp, and is in agreement with recent suggestions that pKsp_BEnamel_ is higher than that reported previously in literature, and may be much closer to the value for pure HAp. However, further similar kinetic studies will be needed to measure enamel solubility at a range of pH conditions, temperatures, and, for example, the influence of salivary proteins, in order to replicate the changing conditions of the oral environment.

## Ethics statement

Written informed consent was obtained from patients who agreed to give their teeth anonymously for research. Ethical approval was granted to use that pool of teeth by Queen Mary Research Ethics Committee (QMREC 2011/99). The site license is in the name of the author FW.

## Author contributions

LH, contribution toward the conception and design of the work. Acquisition, interpretation and analysis of all data. Drafting and revising the work and ensuring the integrity and accuracy of the work. FW, contributions include the interpretation and analysis of data. Revising work and ensuring the integrity and accuracy of the work. RL, contributions include the interpretation and analysis of data. Revising work and ensuring the integrity and accuracy of the work. PA, contributions include the design and conception of the work. Interpretation and analysis of data. Drafting and revising the work and ensuring the integrity and accuracy of the work.

### Conflict of interest statement

Author RL is employed by GlaxoSmithKline and LH is the recipient of a studentship from BBSRC and stipend from GlaxoSmithKline. The other authors declare that the research was conducted in the absence of any commercial or financial relationships that could be construed as a potential conflict of interest.
